# Remodelling of adult cardiac tissue subjected to physiological and pathological mechanical load *in vitro*

**DOI:** 10.1093/cvr/cvab084

**Published:** 2021-03-16

**Authors:** Fotios G Pitoulis, Raquel Nunez-Toldra, Ke Xiao, Worrapong Kit-Anan, Saskia Mitzka, Richard J Jabbour, Sian E Harding, Filippo Perbellini, Thomas Thum, Pieter P de Tombe, Cesare M Terracciano

**Affiliations:** 1 National Heart and Lung Institute, Imperial College London, 72 Du Cane Road, Hammersmith Hospital, ICTEM Building, W12 0NN London, UK; 2 Institute for Molecular and Translational Therapeutic Strategies, Hannover Medical School, OE 8886, Carl-Neuberg-Str. 1, J3 Building, Level 1, Room 3030, 30625 Hannover, Germany; 3 Department of Physiology and Biophysics, University of Illinois at Chicago, 835 S. Wolcott Rm E202 (MC901), Chicago, IL 60612-7342, USA

**Keywords:** Myocardial remodelling, Pressure overload, Volume overload, *In vitro* cardiac tissue culture, Mechanical load, Myocardial slices

## Abstract

**Aims:**

Cardiac remodelling is the process by which the heart adapts to its environment. Mechanical load is a major driver of remodelling. Cardiac tissue culture has been frequently employed for *in vitro* studies of load-induced remodelling; however, current *in vitro* protocols (e.g. cyclic stretch, isometric load, and auxotonic load) are oversimplified and do not accurately capture the dynamic sequence of mechanical conformational changes experienced by the heart *in vivo*. This limits translational scope and relevance of findings.

**Methods and results:**

We developed a novel methodology to study chronic load *in vitro*. We first developed a bioreactor that can recreate the electromechanical events of *in vivo* pressure–volume loops as *in vitro* force–length loops. We then used the bioreactor to culture rat living myocardial slices (LMS) for 3 days. The bioreactor operated based on a 3-Element Windkessel circulatory model enabling tissue mechanical loading based on physiologically relevant parameters of afterload and preload. LMS were continuously stretched/relaxed during culture simulating conditions of physiological load (normal preload and afterload), pressure-overload (normal preload and high afterload), or volume-overload (high preload & normal afterload). At the end of culture, functional, structural, and molecular assays were performed to determine load-induced remodelling. Both pressure- and volume-overloaded LMS showed significantly decreased contractility that was more pronounced in the latter compared with physiological load (*P* < 0.0001). Overloaded groups also showed cardiomyocyte hypertrophy; RNAseq identified shared and unique genes expressed in each overload group. The PI3K-Akt pathway was dysregulated in volume-overload while inflammatory pathways were mostly associated with remodelling in pressure-overloaded LMS.

**Conclusion:**

We have developed a proof-of-concept platform and methodology to recreate remodelling under pathophysiological load *in vitro*. We show that LMS cultured in our bioreactor remodel as a function of the type of mechanical load applied to them.

## Graphical Abstract

**Figure:**
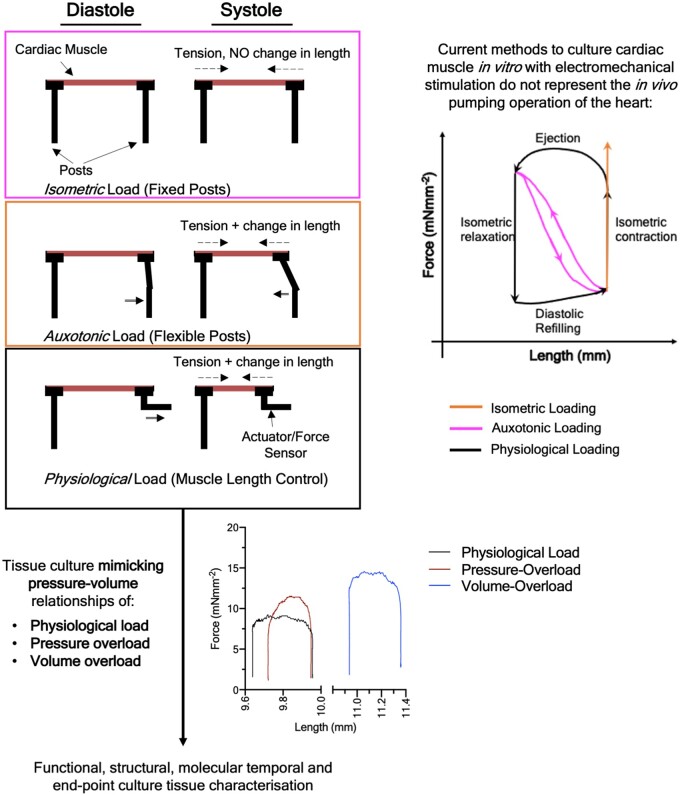

## 1. Introduction

Mechanical load is one of the main drivers of cardiac remodelling, the process by which the heart adapts to physiological or pathological stimuli.^1^ Conventionally, pathological mechanical load has been categorized into pressure- and volume-overload. In the former, excess systolic load or afterload is imposed on the heart, as seen in aortic stenosis or hypertension. In contrast, volume-overload is seen in valvular insufficiency such as mitral regurgitation and is characterized by pathologically high preload. Although mechanical overload has been studied extensively *in vivo*^2^^,^^3^; it remains less well characterized using *in vitro* cardiac models.

**Figure 1:**
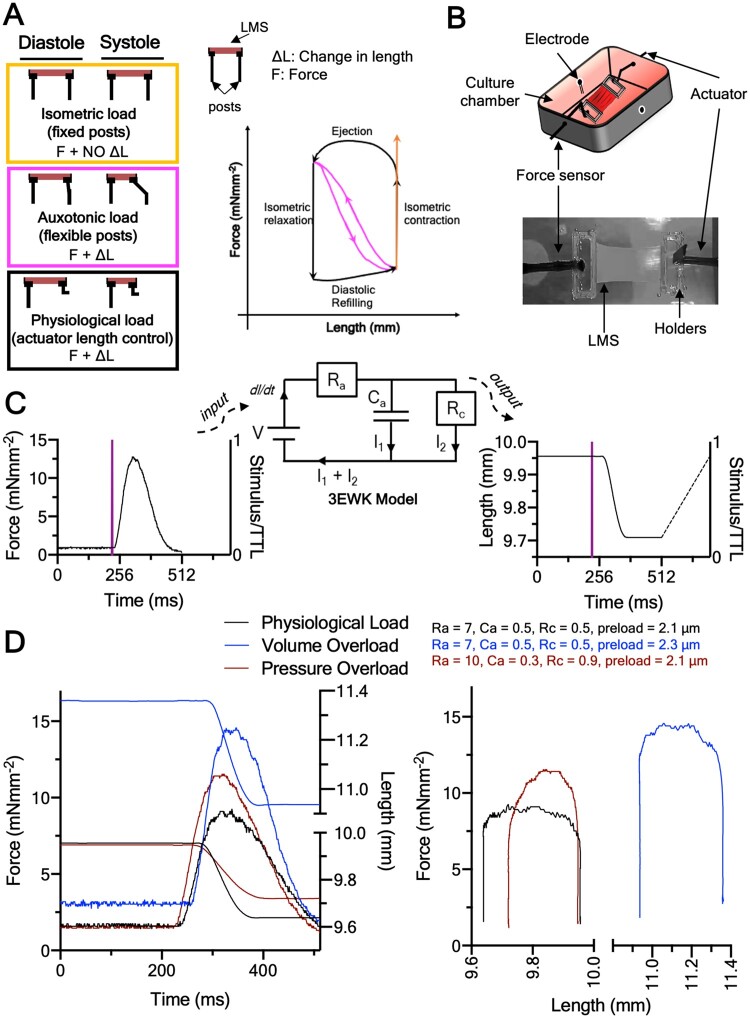
Cardiac tissue electromechanical culture and principle of operation. (*A*) Current methods to culture cardiac tissue under electromechanical stimulation *in vitro*. *Top panel*: isometric culture against fixed posts. *Middle panel*: auxotonic contraction against flexible posts. *Bottom panel*: actuator control of length based on force output of tissue—the methodology used here. These culture methods are transposed color-coded on the force-length plane. (*B*) Cartoon schematic and image of an LMS suspended in our custom bioreactor. (*C*) Operation of bioreactor. Force transients are acquired on each beat and fed to a custom LabVIEW program running the 3EWK model. The bioreactor then actuates the length-waveform predicted by the 3EWK on the LMS. (*D*) Representative force-length transients and loops of the LMS in the different loading groups, and the preload and afterload used for each. Note that the diastolic phase, although the same for all groups, was not acquired, and therefore loops appear as open bottom.

*In vitro* models allow for a reductionist representation of the *in vivo* state and are indispensable for basic and pre-clinical research.^4^ Currently, the effects of chronic load are studied *in vitro* by stretching cardiac tissue during culture. The most common approach is to mount cardiac muscle on flexible posts which bend upon force development.^5^^,^^6^ This provides an auxotonic systolic load that can be increased or decreased by changing the stiffness of the posts, while preload is set by stretching the tissue to the desired diastolic length (*Figure 1A*). In other approaches, tissue is cultured on fixed posts where it contracts isometrically—that is, under effectively infinite, and therefore unphysiological afterload^7^^,^^8^ (*Figure 1A*), or cyclic stretch.^9^

Under such culture methods, the dynamic mechanical events of the *in vivo* cardiac cycle comprising the distinct phases of isometric contraction, ejection, isometric relaxation, and diastolic refilling are not recreated. For example, auxotonic load, the current gold-standard, has a linear length–force relationship akin to that of an elastic band (*Figure 1A*). This limits the physiological relevance and translation of findings.^4^

We developed a platform to recreate the *in vivo* physiological or pathological mechanical load of the heart on living myocardial slices (LMS) *in vitro* for extended periods of time (*Figure 1A and B*). LMS are 300 μm thick organotypic living sections of heart tissue. Their thinness allows for oxygen diffusion,^10^ while their structure (multi- and heterocellularity, extracellular matrix [ECM], and cell stoichiometry), and function (contractility, electrophysiology, and metabolism) match that of the *in-situ* heart,^4^ making them an intermediate-complexity cardiac model. For a detailed review on the LMS model, see Ref.^4^

We cultured adult rat LMS for 3 days in a custom bioreactor designed to subject them to the *in vivo* electromechanical pressure–volume relationships. To do that, we used a 3-Element Windkessel (3EWK) mathematical model that worked in sync with electrical stimulation to mechanically load LMS (*Figure 1C*). The 3EWK describes afterload in terms of arterial impedance (Ra), compliance (Ca), and peripheral resistance (Rc), all of which have physiological relevance,^11^ while preload was set by manipulation of sarcomere length (SL). This approach allowed us to parameterize both afterload and preload and study the effects of distinct mechanical conditions on LMS. Three mechanical load profiles were examined: physiological load, pressure-overload, or volume-overload (*Figure 1D* and see Supplementary material online, *Figure S2*). We performed functional, structural, and molecular characterization and show that the remodelling response of adult myocardium to pathological mechanical load can be recreated and studied *in vitro*.

**Figure 2:**
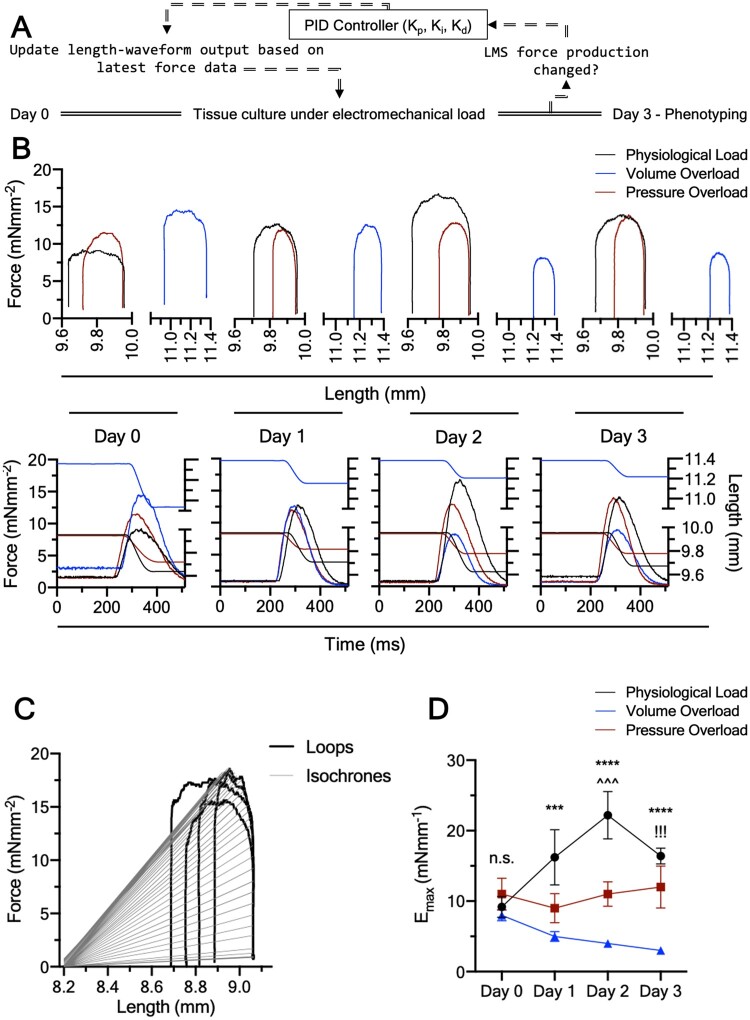
Adult cardiac tissue culture under adaptive electromechanical stimulation. (*A*) Algorithm used by the bioreactor during the 3 day culture. Each force-transient produced by LMS was sampled and fed to the 3EWK model. If the force changed the length-transient performed on the LMS adapted to reflect that based on a PID algorithm. This ensured the bioreactor was always adapting to the remodelling tissue. (*B*) Representative 3 day force-length loops and force-length transients for the LMS cultured under physiological load, pressure-overload, or volume-overload. (*C*) Modified time-varying elastance for assessment of contractile state. Grey straight lines are isochrones constrained at the LMS’s RL (∼8.2 mm in graph). Only a fraction of the isochrones is plotted for clarity. (*D*) Maximum elastance, *E*_max_, corresponding to load-independent intrinsic contractile state for the three groups across time. *Note*: All LMS start at the same *E*_max_. Data shown as mean ± SEM; (N = 6). *P*-value was calculated using two-way ANOVA with Tukey’s multiple comparison test.

## 2. Methods

### 2.1 Bioreactor development

A bioreactor that could recreate the cardiac cycle *in vitro* was developed. The bioreactor consisted of a 300C dual-mode muscle lever (Aurora Scientific, Ontario, Canada), which can simultaneously measure force and lengthen/shorten tissue, and a custom CNC-machined culture chamber (Ertacetal-C), which accommodated the LMS, media, perfusion inlets/outlets, and electrodes for tissue pacing (*Figure 1B*). The LMS was mounted between the lever of the 300C and an inflexible post and stimulated to contract via the electrodes at 1 Hz using a bipolar constant voltage pacer (IonOptix, MA, USA; *Figure 1C*). Culture media was recirculated using a MasterFlex pump (Cole-Parmer, Eaton, UK), and the whole set-up was maintained at 37°C using an incubator.

The bioreactor sampled 512 ms of each LMS force transient at 1 kHz frequency following each TTL stimulation trigger pulse. Force data were acquired every 5 min for the whole of the 3 day culture (see Supplementary material online, *Figure S1*) and the first data were logged after the LMS had been on the bioreactor for 15 min. Each force transient was converted to pressure using Laplace’s law, and used as input to a 3EWK mathematical model. In the electrical analogy of the 3EWK, current (I) corresponds to blood flow, voltage (V) to pressure, and *dI/dt* to the rate of change of ventricular volume (*Figure 1C*). The 3EWK was numerically solved in the pressure and volume state-space and the predicted volume waveform was converted to tissue length based on a spherical model of the left ventricle (LV).^12^ This was then iteratively applied to the beating LMS in sync with the electrical stimulation using a proportional-integral-derivative (PID) algorithm as previously described^12^ (*Figure 2A*). The diastolic phase was set to a linear ramp actuated within 200 ms after the end of 512 ms data acquisition. Data from the diastolic phase were not acquired, and thus pressure–volume loops are shown with an open bottom (*Figures 1C and 2B*).

Ultimately, this allowed the LMS to continuously ‘loop’ during culture, shortening in length while producing force (systole) and stretching back to diastolic length during relaxation (diastole), recreating cardiac work loops (*Figures 1A–D and 2A and B*).

As the length-waveform predicted by the 3EWK depends on the force generated by the LMS, and the process of force acquisition, length-waveform prediction, and actuation were performed for each beat for the whole culture (∼259 200 beats for the 3-day culture), the LMS were always beating based on the latest force data, making the bioreactor adaptive to the remodelling tissue. All software was programmed in LabVIEW (National Instruments, TX, USA).

### 2.2 LMS preparation

All animal experiments were conducted in accordance with institutional and national regulations, and approved by Imperial College London, under license by the UK Home Office, United Kingdom Animals (Scientific Procedures) Act 1986 Amendment Regulations 2012, and EU directive 2010/63/EU.

LMS were prepared according to Ref.^10^ Sprague–Dawley rats were euthanized under isoflurane-induced anaesthesia (4% isoflurane at 4 L/min oxygen) by cervical dislocation. The heart was quickly explanted, and placed for 5 s in a 60 mL vial containing 37°C modified heparinized (1000 IU/mI) Tyrode solution (30.0 mM 2,3-Butanedione Monoxime, 140.0 mM NaCl, 9.0 mM KCl, 10 mM Glucose, 10.0 mM HEPES, 1.0 mM MgCl_2_, 1.0 mM CaCl_2_ at pH of 7.40). This allowed any residual blood inside the ventricles to be pumped out, facilitating the subsequent dissection. The heart was then placed inside a 60 mL vial containing 4°C heparinized modified Tyrode solution (same as above) and kept there until dissection.

The vial containing the heart was emptied into a petri dish and the lungs, trachea, and non-cardiac tissue removed using a scalpel. To isolate the LV, the right ventricle (RV) was dissected and disposed with the use of forceps and microscissors. The interventricular septum was then cut across using microscissors allowing the LV to be propped open and flattened by cutting the papillary muscles. The LV was glued epicardial face down to agar on a specimen holder using histoacryl glue (Braun, DE), and the specimen holder mounted to the organ bath of a vibrating microtome (7000 smz2, Campden Instruments Ltd, Leicester, UK). The vibratome was previously calibrated to *z*-axis <1 μm, and the organ bath filled with oxygenated 4°C modified Tyrode solution. Once the organ bath was mounted the slicing procedure was started with a 0.03 mm/s vibratome advance speed, 80 Hz frequency, and 2 mm amplitude. One LMS was generated at a time every 5–10 min. As the ventricular wall exhibits heterogeneity in its structural and functional properties^13^ only mid-myocardial LMS were used for the experiments.

The LMS produced by the vibratome were examined under light microscopy to determine myocardial fibre orientation, and their length and width recorded with callipers for normalization of force to the cross-sectional area. Custom 3D-printed polylactic acid holders of rectangular shape (12 × 3 × 0.5 mm; as shown in *Figure 1B*) were glued to the ends of each LMS perpendicular to the main fibre axis using histoacryl glue. The holders allowed physical manipulation of the LMS and mounting to the bioreactor.

### 2.3 LMS culture

LMS were picked up from their holders using forceps, mounted on the bioreactor chamber, and stretched to the desired preload using callipers and muscle length as surrogate for SL. The LMS were allowed to beat for 15 min before the 3EWK length-waveforms were actuated and the data were first logged. For physiological load LMS were stretched to a normal preload (110.5% of resting length (RL), corresponded to 2.1 SL—see Supplementary material online, *Figure S2*) and intermediate 3EWK parameters (Ra = 7, Ca = 0.5, Rc = 0.5), chosen based on their ratios, which reflect nominal afterload.^14^ The pressure-overloaded LMS were stretched to the same preload but with high aortic impedance (Ra = 10), low aortic compliance (Ca = 0.3), and high peripheral resistance (Rc = 0.9), to model high afterload. For volume-overload, the LMS were stretched to a pathologically high preload (126.3%, corresponded to 2.3 μm SL—see Supplementary material online, *Figure S2*) but with physiological afterload (same as physiologically loaded LMS; Ra = 7, Ca = 0.5, Rc = 0.5).^8^ When these load profiles were first imposed on the LMS at the beginning of culture, the work loops performed by each group closely mimicked the expected acute *in vivo* pressure–volume trajectories of the corresponding load condition^2^ (*Figure 2B*). After starting the work loop algorithms, the LMS were kept in culture for 3 days.

Media-199 (Sigma-Aldrich, MO, USA) supplemented with rat basal plasma concentrations of noradrenaline (4 nM), adrenaline (4 nM), triiodothyronine (2.15 nM), dexamethasone (100 nM), and l-ascorbic acid (0.002 g/mL; Sigma-Aldrich, MO, USA) was used for culture. The addition of hormones aimed at providing a physiological humoral environment and was kept the same in all groups. The entire superfusate was replaced every day with fresh media. All LMS were field-stimulated at 1 Hz with 5–10 ms, 5–10 V square pulses. 95% O_2_, 5% CO_2_ (BOC, UK) was used for oxygen supplementation.

Culture and the experiments described in 2.4 and 2.5 were conducted while the bioreactor was inside an incubator at 37°C, 95% O_2_, 5% CO_2_.

### 2.4 Assessment of time-varying elastance (*E*_max_)

On each day of culture, all LMS were made to do four work loops with the same 3EWK parameters. This was done to determine the load-independent intrinsic contractile state of the LMS (time-varying elastance). The four work loops had 3EWK parameters that covered a spectrum of systolic loads (1. Ra = 7, Ca = 0.5, Rc = 0.5, 2. Ra = 5, Ca = 0.7, Rc = 0.2, 3. Ra = 10, Ca = 0.3, Rc = 0.9, and 4. Ra = 5, Ca = 0.5, Rc = 0.5; *Figure 2C*) and were fed to a custom LabVIEW algorithm (see Supplementary material online, *Figure S3*), which accepted any number of work loops and outputted a sequence of isochrones and the maximum elastance, *E*_max_. As force and length were used instead of pressure and volume the time-varying elastance was modified such that dead volume, *V*_d_, of the original theory corresponded to resting muscle length, that is the muscle length (or volume in the original theory) below which no force (or pressure in the original theory) generation occurs. The rationale behind this approach is explained in Section 3.2.

**Figure 3:**
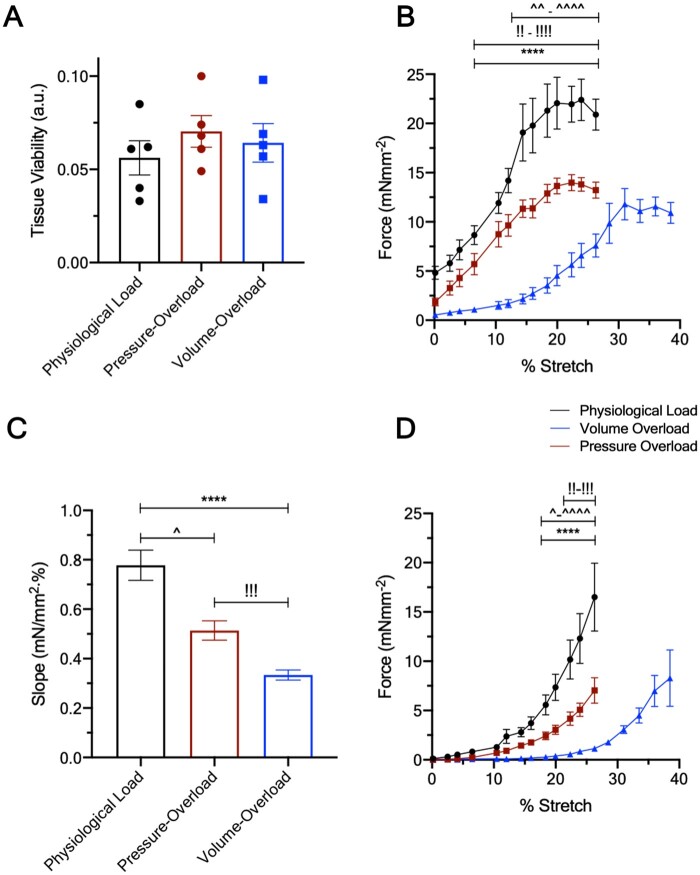
Viability and functional remodelling of cultured LMS. (*A*) Tissue viability of cultured LMS (*N* = 5). (*B*) Active Frank–Starling relationship of cultured LMS. (*C*) Slopes of the force–% stretch relationship of the cultured LMS. (*D*) Passive Frank–Starling relationship of cultured LMS. Data are shown as mean ± SEM; *N* = 6 for physiological and volume-overload, *N* = 4 for pressure-overload. *P*-value was calculated using two-way ANOVA with Tukey’s multiple comparison and ANCOVA for force–% stretch relationship and slopes of linear regression, respectively.

### 2.5 Frank–Starling curves and linear regression

Isometric Frank–Starling experiments were performed as end point culture assays at 1 Hz stimulation. LMS were stretched from RL to 126.3% in physiological and pressure-overload and 138.5% in volume-overload. The higher stretch in the latter group was the result of preliminary work indicating that peak isometric force was not reached at 126.3% of RL (*Figure 3B*). All analysis was done using custom LabVIEW codes that automatically determined the amplitude, baseline, and kinetics of the acquired data.

The sensitivity of LMS to stretch was analysed by fitting a linear regression along with the force–stretch relationship and comparing the slopes (*Figure 3C*).

### 2.6 Viability assays

The viability of cultured LMS was assessed using CellTiter 96 Aqueous One solution cell proliferation assay (Promega, Southampton, UK), as previously described.^8^ A 2 mm diameter sample was obtained from the cultured LMS using a biopsy puncher. The sample was incubated for 20 min at 37°C, 95% O_2_ 5% CO_2_ in a 96-well plate filled with 100 μL M-199 + 40 μL CellTiter96. The absorbance of the media in the well was then recorded immediately at 490 nM using a 96-well plate reader (Labtech, TX, USA). Each value was normalized by subtracting the background (incubated M-199 + CellTiter96, no LMS) absorbance.

### 2.7 Tissue media pH

After removing the LMS from the bioreactor on the last day of culture, the pH of the culture media bathing the LMS was measured using a pH meter (Mettler Toledo, OH, USA; see Supplementary material online, *Figure S4*).

**Figure 4:**
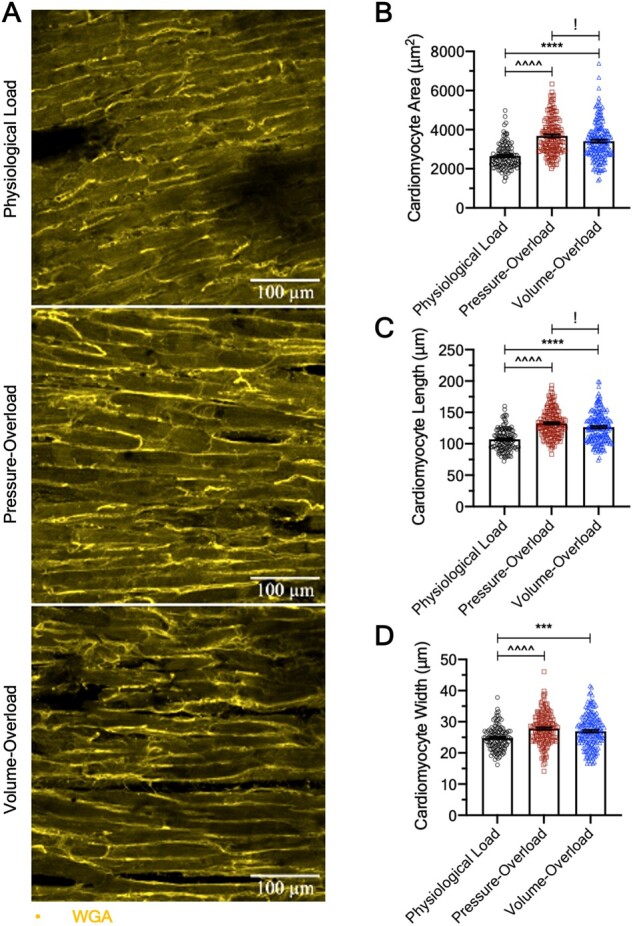
Structural remodelling of cultured LMS. (*A*) ×20 representative confocal images of WGA-stained cultured LMS for assessment of cardiomyocyte dimensions. (*B*) Cardiomyocyte area. (*C*) Cardiomyocyte length. (*D*) Cardiomyocyte width. All data were analysed by two independent blinded reviewers and shown as mean ± SEM; *N* = 120/3, 159/4, and 184/5 cells/biological replicates for physiological, pressure- and volume-overload, respectively. One-way ANOVA with Tukey’s multiple comparison was used for calculation of *P*-value.

### 2.8 Confocal imaging and analysis

Three-day cultured LMS were removed from the bioreactor, mounted on custom-made stainless-steel stretchers, stretched to 110.45% stretch, and fixed in 4% formaldehyde for 15 min at room temperature. They were then washed in PBS, permeabilized, and blocked using 1.5% Triton X-100 in 10% FBS, 5% BSA, and 10% horse serum for 3 h at room temperature on a rocker. After washing, LMS were incubated with wheat germ agglutin (WGA) for 15 min and washed again three times with PBS.

Stained LMS were visualized under confocal microscopy (Zeiss LSM-780, DE) at ×20 magnification. Z-stacks were obtained until the thickness of the tissue made further visualization impossible. Five images per LMS were taken.

Confocal image analysis was performed blinded by two independent reviewers in ImageJ (NIH, MD, USA). For cardiomyocyte morphology, a rectangle fitting the largest edges of a cardiomyocyte was drawn for 8 cardiomyocyte per image (see Supplementary material online, Figures S5 and S6). The length and height of the rectangle were aligned along the length and width of the cardiomyocyte, respectively. The area of the rectangle and the length/width were then measured to determine cardiomyocyte area, length, width, and aspect ratio.

**Figure 5:**
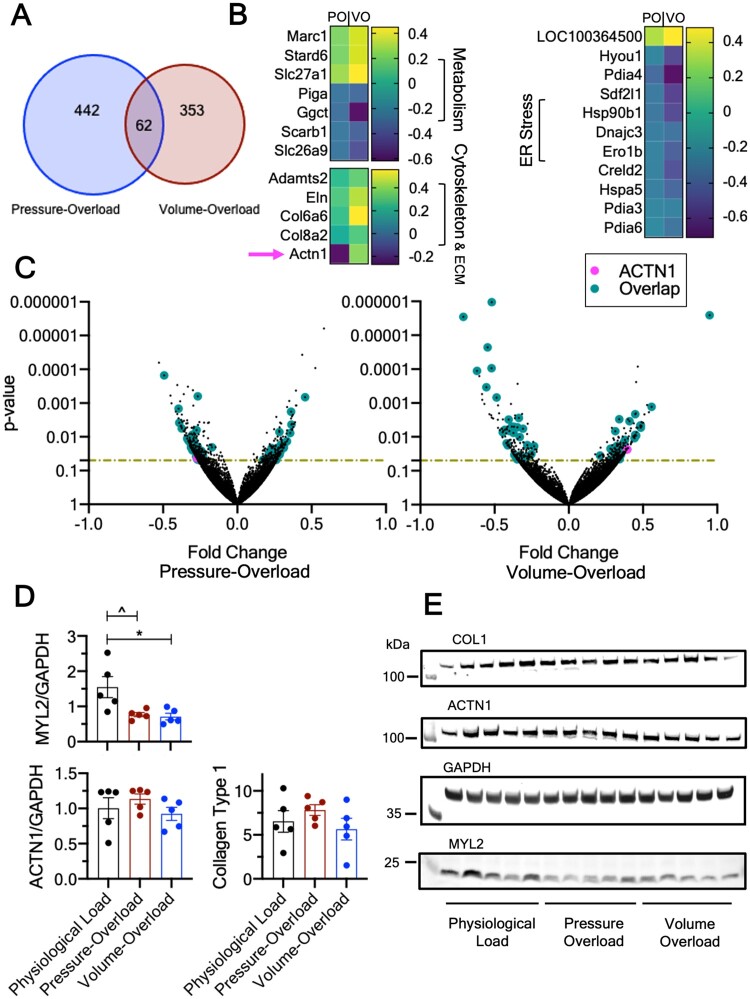
Transcriptomic and protein signatures of cultured LMS. (*A*) Venn diagram of number of significantly (*P* < 0.05) differentially regulated genes in pressure- overload and volume-overload. (*B*) Heatmaps of selected genes that were differentially regulated and shared by both overloaded groups. *Actn1* is marked with an arrow as it showed polarized direction of expression. (*C*) Volcano plots of the pressure- and volume-overloaded differentially regulated genes. Horizontal lines denote *P* = 0.05. Only genes above this line were considered for pathway analysis and interpretation of findings. Teal-coloured points denote commonly shared genes that show same direction of expression in the overloaded groups. Pink-coloured points show *Actn1* which showed polarized direction of expression. (*D*) Western blots for MYL2, ACTN1, and Collagen Type 1, and (*E*) Representative gels. For RNAseq, *N* = 4 and *P* values were determined using Fischer Exact test. For western blots, *N* = 5 and *P* values determined using One-way ANOVA with Tukey’s multiple comparison. Data are shown as mean ± SEM.

**Figure 6:**
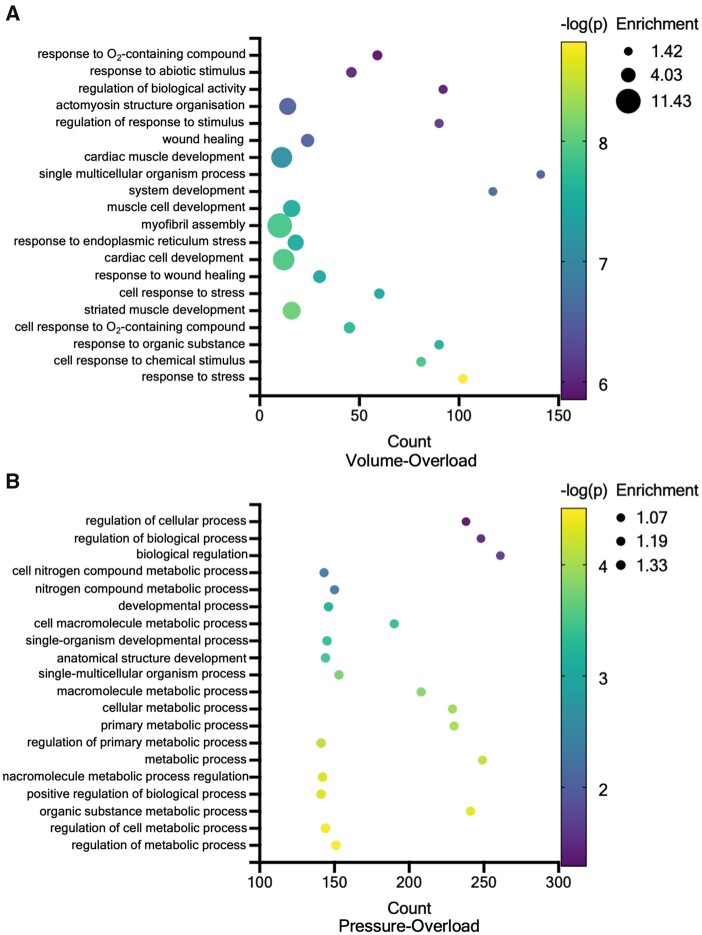
Functional enrichment analysis of transcriptomic signatures. (*A*) Gene ontology analysis of volume-overloaded cultured and (*B*) pressure-overloaded LMS.

### 2.9 Western blots

Cultured LMS were removed from the bioreactor, quickly washed in PBS, patted dry, snap-frozen in liquid N_2_, and stored at -80°C. LMS were mechanically disrupted and protein was extracted using a TissueLyser LT and 5 mm stainless steel Beads (Qiagen, Hilden, DE) in Eppendorfs containing 300 μL of RIPA buffer completed with protease and phosphatase inhibitor pills (Thermofisher, MA, USA). Protein concentration was determined using the Pierce BCA Assay kit (Thermofisher, MA, USA) by following the manufacturer’s instructions. Then, 30 μg of protein and 5 μL: of 4X Bolt™ LDS sample buffer (Thermofisher, MA, USA) with 5% of 2-mercaptoethanol were mixed (total volume = 25 μL), loaded on a 17-well Bolt™ 4–12% SDS-PAGE gel (Thermofisher, MA, USA) filled with SDS Bolt™ Running Buffer (Thermofischer, UK) and run for 25 min at 200 V. Proteins were transferred to 0.45 μΜ PVDF transfer membranes (Sigma-Aldrich, MO, USA) with Bolt™ Transfer Buffer (Termofisher, UK) and probed with GAPDH (rabbit, 1:1000; Cell Signaling, MA, USA), collagen type I (rabbit, 1:1000; Abcam, Cambridge, UK), α-actinin (mouse, 1:1000, Abcam, Cambridge, UK), myosin light chain 2 (rabbit, 1:1000, Abcam, Cambridge, UK) and myosin heavy chain (rabbit, 1:800, Abcam, Cambridge, UK) at 4°C on a rocker overnight. Bands were detected with BIORAD ChemiDoc Touch Imaging system (BIO-RAD, UK) using IRDye 800CW donkey anti-mouse and IRDye 680RD donkey anti-rabbit (1:2000; LI-COR Biosciences, UK). Images were analysed in ImageJ. With the exception of type 1 collagen, all other proteins were normalized to GAPDH. This is because fibrosis relates to the total amount of collagen.

### 2.10 RNA extraction and sequencing

Both sample processing and analysis were done by two separate blinded investigators. After the culture, LMS were snap-frozen in liquid N_2_. A tissue homogenizer, Precellys 24 (Bertin Instruments, Montigny-le-Bretonneux, FRA) was used to disrupt the sample. The frozen LMS were transferred to a 2 mL Micropackaging vial containing Precellys Ceramic Beads 2.8 mm and 700 μL of Qiazol (Qiagen, DE). The Precellys was set to 2 × 20 s at 5500 rpm. RNA purification was performed with the miRNeasy Mini Kit (Qiagen, DE) according to the manufacturer’s instructions. The RNA was frozen and stored at -80°C until next-generation sequencing.

Then, 500 ng of total RNA per sample were utilized as input for rRNA depletion procedure with ‘NEBNext^®^ rRNA Depletion Kit (Human/Mouse/Rat), 96 rxns’ (E6310X; New England Biolabs, MA, USA) followed by stranded cDNA library generation using ‘NEBNext^®^ Ultra II Directional RNA Library Prep Kit for Illumina’ (E7760L; New England Biolabs). Sequencing was performed on an Illumina NextSeq 550 sequencer using a High Output Flowcell for 2 × 75 bp paired-end reads. BCL files were converted to FASTQ files using bcl2fastq v2.20.0.422 (Illumina, San Diego, USA).

Raw reads were aligned to rat genome reference (rn6.0) utilizing RNA-STAR, and annotations taken from iGenomes (Rattus_norvegicus/Ensembl/Rnor_6.0). Reads counts normalization and differential expression analysis were performed with DESeq2 (default settings). Normalized read counts were then used for hierarchical clustering applying Cluster3. Functional annotation and enrichment of the significantly regulated genes were performed in DAVID version 6.8 with integrated GO database. Minimum number of genes was set to 2 for corresponding pathway; maximum EASE score (*P*-value of Fisher Exact test) was set as 0.05 for a significant match.

### 2.11 Laser diffraction experiments

LMS were mounted on custom-made stretchers and positioned inside a glass dish, filled with modified Tyrode’s solution at room temperature. A HeNe laser (Lasos, Jena, DE) 2 cm vertically above the slice was turned on. The passing light diffracted onto bands as a function of tissue stretch. A C920 HD camera (Logitech, Lausanne, CH) captured and analysed the diffraction pattern in ImageJ in real-time. LMS were stretched from RL to three different % stretches and the SL at each stretch acquired. A linear regression was fit to the % stretch-SL data. From this, and given the RL of an LMS, the % stretch required to attain the desired SL from RL was calculated and used to set the preload at the beginning of culture (see Supplementary material online, *Figure S2*).

### 2.12 Statistics

Statistical analysis was performed in Prism 8.0 (GraphPad, San Diego, USA). Data were first analysed for normal distribution by visual inspection and then using the D’Agostino–Pearson normality test. For normally distributed sets of data one-way and two-way analysis of variance (ANOVA) with Tukey’s multiple comparisons test was used to determine whether there were any statistically significant differences between the means of groups. For sets of data that were not normally distributed, Kruskal–Wallis test was used to determine statistical significance between the means of groups. For the statistical analysis of the slopes from the force and % stretch linear regressions, analysis of covariance (ANCOVA) was used. *P* < 0.05 was considered statistically significant.

*, **, ***, ****: *P* < 0.05, *P* < 0.01, *P* < 0.001, and *P* < 0.0001 physiological load vs. volume-overload.

^, ^^^, ^^^^^^, ^: *P* < 0.05, *P* < 0.01, *P* < 0.001, and *P* < 0.0001 physiological load vs. pressure-overload.

!,!!,!!!,!!!!: *P* < 0.05, *P* < 0.01, *P* < 0.001, and *P* < 0.0001 volume-overload vs. pressure-overload.

## 3. Results

### 3.1 LMS culture under dynamic and tissue-responsive electromechanical stimulation

Upon mounting on the bioreactor, the force produced by an LMS was acquired and fed to the 3EWK model. Thus, for each force transient a corresponding length-transient was outputted, the characteristics of which were determined by the input force transient and the chosen 3EWK parameters (Ra, Rc, and Ca). The length-transient was then actuated on the LMS. As the model run continuously throughout culture based on a PID feedback algorithm, a change in LMS force development was accompanied by a change in the length-transient, meaning that the bioreactor was adapting to the remodelling LMS (*Figure 2A and B*). For example, volume-overloaded LMS showed a monotonic decline in force production from Day 0 to Day 3 and this was reflected by a decreasing stroke length (the length by which the tissue is shortening) in the length plane. Ultimately, this feedback-based approach enabled iterative mechanical loading of LMS equivalent to more complex dynamic systems like *in vivo*, where LV volume is a function of contractility.^1^

### 3.2 Load-induced contractile remodelling

Because of the different experimental loading conditions, LMS in each culture group were contracting against different systolic and diastolic loads. This means that twitch force, which depends on load, could not be used as a reliable measure of contractility.^15^ For example, at high preload and physiological afterload, the volume-overloaded LMS produced a greater twitch force than the physiologically loaded LMS on Day 0 (*Figure 2B*). Likewise, with equal preload but higher afterload the pressure overload LMS produce a greater twitch force than physiologically loaded LMS (*Figure 2B*). This does not reflect stronger LMS but rather LMS contracting against different loads.

To solve that we developed an iterative algorithm (see Section 2 and Supplementary material online, *Figure S3*) to calculate the time-varying elastance, *E*_max_. *E*_max_, which is used clinically,^16^ considers both the length and twitch force of a series of loops (*Figure 2C*), and is a load-independent measure of intrinsic contractility. In support of the superiority of this approach, although the twitch force of the groups was different at Day 0, the *E*_max_ was not (Figure *2D*—Day 0), as would be expected from freshly prepared tissue not yet subjected to culture.

The physiologically loaded LMS were significantly stronger at Day 2 compared to their Day 0 counterparts, suggesting temporal fluctuations in their *E*_max_. From our experience, LMS undergo an initial ‘acclimating’ phase characterized by a rapid decline in force production during the first hours in the bioreactor (see Supplementary material online, *Figure S1*). This is most likely a result of the LMS method of preparation, which involves contraction uncouplers and a high K^+^ solution (see Section 2 and Ref.^4^) and leads to a hampered and thus underrepresented force production at Day 0.

More importantly, although all groups start at the same *E*_max_ at Day 0, LMS cultured under volume-overload were significantly weaker than physiologically loaded (Day 1–Day 3) and pressure-overloaded (Day 3) LMS. This can be appreciated by work loops of decreasing width and height suggesting that pathological preload is more damaging to the contractile phenotype than pathological afterload within the timeframe examined here. In contrast, pressure-overloaded LMS showed a preserved *E*_max_ that did not decline with culture (*Figure 2D*). There were no significant differences in tissue viability suggesting that these findings are not underlined by tissue death (*Figure 3A*).

To elucidate the contractile reserve of LMS and their ability to respond to stretch, a physiological stressor, isometric force–stretch experiments were conducted at end-point culture. All groups showed a positive Frank–Starling relationship, yet each had a unique response to stretch (*Figure 3B–D*).

Pressure-overloaded and physiologically loaded LMS had similar trajectories but the former were unable to attain the same absolute force at the highest stretch (116–126%; *Figure 3B*). Furthermore, the force–% stretch slope of pressure-overloaded LMS, calculated by fitting a linear regression to the data, was significantly decreased (*Figure 3C*). These findings suggest that despite a preserved intrinsic contractility (*E*_max_), LMS cultured under pressure-overload have an inadequate response to acute mechanical stress.

Volume-overloaded LMS showed a rightward shift in their force–stretch response, with significantly decreased force generation across the stretch range (110–138%; *Figure 3B*) and force output per % stretch (*Figure 3C*) compared to physiologically loaded and pressure-overloaded LMS. Together with the decreased *E*_max_, this point towards greater and almost global systolic failure.

Although the distensibility (Δstretch/ΔForce) of both pressure- and volume-overloaded LMS was significantly higher than that of physiological load, it was more severely affected in volume-overloaded LMS suggesting alterations in passive mechanics akin to those seen in dilated cardiac phenotypes (*Figure 3D*).^17^

### 3.3 Load-induced structural remodelling

Cardiomyocyte area, width, and length were analysed in fixed LMS stained with the membrane marker WGA by two independent blinded investigators and found to be significantly increased in pressure- and volume-overloaded LMS (*Figure 4A–D*). Thus, both overloaded groups show hypertrophic phenotypes with features of concentric (increased cardiomyocyte width) and eccentric (increased cardiomyocyte length) remodelling. The pressure-overloaded LMS also had significantly greater increases in their cardiomyocyte size and length compared to volume-overloaded LMS, suggesting a greater hypertrophic response.

### 3.4 Load-dependent molecular remodelling

The transcriptomic and proteomic profile of cultured LMS was investigated using blinded RNAseq and western blots. The expression of 442 and 353 genes was significantly different in pressure- and volume-overload compared to physiological load (*Figure 5A*), with 62 genes shared between the two overloaded groups. The remaining 380 and 291 differentially regulated genes were unique to either overloaded condition, and correspond to the black dots above the *P*-value horizontal line (*Figure 5C*). Of the 62 overlapping genes, the endoplasmic reticulum stress response pathway was enriched, while multiple genes involved in metabolism, cytoskeleton, and ECM remodelling were also significantly altered (*Figure 5B*). Interestingly, 61/62 shared genes showed the same direction of expression (teal coloured dots, *Figure 5C*) and only 1/62 genes (*Actn1*, pink coloured dots, *Figure 5C*) had polarized expression, being significantly down-regulated in pressure- and up-regulated in volume-overload.

Analysis of the individual groups revealed that pathways involved in cardiac muscle development (GO: 0055013, GO: 0055001, GO: 0055006, GO: 0055002) and sarcomeric organization and assembly (GO: 0030239, GO: 0031032) were explicitly enriched in volume-overloaded but not pressure-overload LMS (*Figure 6A*). Instead, the latter showed enrichment of pathways associated with response to inflammation and stress (GO: 0050794), and metabolism (GO: 0080090, GO: 0031323; *Figure 6B*).

Western blots showed that myosin light chain 2 (MYL2) was significantly down-regulated in both pressure- and volume-overload (*Figure 5D and E*). Despite the gene expression differences in *Actn1* there were neither significant differences in its protein levels, nor in the expression of type 1 collagen, and myosin heavy chain (*Figure 5D and E* and Supplementary material online, *Figure S6*).

## 4. Discussion

We cultured adult rat LMS under physiological, pressure-, or volume-overload for 3 days using a custom bioreactor that simulated afterload based on a 3EWK model and preload based on SL. Both overloaded groups had preserved viability but showed decreased force production upon stretch, cardiac hypertrophy, and transcriptomic and proteomic dysregulation with genes that were both shared and unique to each group.

### 4.1 State of the art electromechanical cardiac muscle tissue culture

*In vitro* culture of cardiac muscle is an indispensable tool for cardiovascular research. It allows greater experimental control than more complex whole-organism systems and often higher experimental throughput.^4^ There are numerous cardiac muscle models including isolated cardiomyocytes, papillary muscles, engineered heart tissues (EHTs), and living myocardial slices (LMS).^4^ The aim of this project was to study remodelling on adult cardiac tissue *in vitro*. As such, we used LMS, an intermediate complexity organotypic model that retains the intact structure, function, and molecular signatures of the *in situ* heart.

In contrast to other culture methods from ours^8^ and other labs^6^^,^^7^^,^^9^ which either do not load, or use rudimentary loading protocols (auxotonic-, isometric-, or cyclic stretch) during culture, here we recreate the *in vivo* cardiac cycle with relevant parameters of afterload (Ra—aortic impedance, Rc—peripheral resistance, and Ca—aortic compliance) and preload (sarcomere length). Although the 3EWK has long been used for acute cardiac studies,^12^ to the best our knowledge, this is the first time it has been used in chronic *in vitro* culture.

In the past we^18^ and recently others^19^ have performed work-loops *in vitro* by applying pre-calculated length waveforms based on offline force data (i.e. not real-time). This is not physiologically correct in the study of remodelling. Specifically, changes in force production in a remodelling tissue must be reflected by corresponding adjustments in length for the same afterload/resistance to be imposed on the tissue. The method described herein addresses that by employing a close feedback-loop.

### 4.2 Remodelling as a function of mechanical load

A central tenet in cardiac remodelling is that the heart hypertrophies to normalize wall stress according to LaPlace’s law.^20^ Conventionally, pressure-overload results in concentric hypertrophy (increased LV thickness and cardiomyocyte width) while volume-overload causes eccentric hypertrophy (increased LV radius and cardiomyocyte length).^1^ However, this binary classification may be too simplistic.^1^ For example, even in the purest forms of pressure-overload, increases in cardiomyocyte size and width are often accompanied by cardiomyocyte elongation.^17^^,^^21–23^ Likewise, LV internal diameter and wall thickness can both increase in volume-overload^24^ and increased myocyte width has been shown in volume-overloaded mice.^2^ Additionally, an increase in LV thickness is necessary for the excess blood filling the ventricle to be pumped.^2^ We found that both pressure- and volume-overloaded LMS increased in size, length, and width compared to physiologically loaded LMS, although the first did so to a greater extent. It is likely that this is due to a weaker hypertrophic response from diastolic than systolic wall stress.^25^

Both overloaded groups showed decreased contractile performance. In volume-overload, this was global and similar to that seen in overt systolic dysfunction.^16^^,^^26^ In pressure-overload the capacity to respond to mechanical stress was decreased with force-stretch relationships similar to those seen *in vivo* during pressure-overload induced hypertrophy of the cat RV and isolated working hearts from spontaneously hypertensive rats.^27^^,^^28^ In both groups, these findings were associated with significantly decreased MYL2 protein expression, an essential contractile sarcomeric protein, the absence of which causes embryonic lethality and sarcomeric malformations.^29^^,^^30^

Ultimately, we found that pathological preload was associated with more severe contractile dysfunction than pathological afterload. Findings regarding this have been controversial in the literature. Carabello *et al*. showed greater depression in contractility in mitral regurgitation (a state of volume-overload) as opposed to aortic stenosis in dogs.^31^ In contrast, TAC-induced pressure-overload is more deleterious than aortocaval shunt-induced volume-overload in mice.^2^

Interestingly, the volume-overloaded LMS had a pronounced drop in their force-% stretch slope as well as a rightward shift in this relationship. This means that (i) volume-overloaded LMS require a supra-physiological amount of stretch to maintain force output, and that (ii) the working stretch range to which the muscle is responsive is critically diminished. *In vivo*, a decrease in cardiac output activates a sequela of neurohormonal pathways including the adrenergic and renin–angiotensin–aldosterone system.^32^ Simultaneously, the decreased perfusion leads to lower capillary hydrostatic pressure and absorption of interstitial fluid into the circulation, governed by basic Starling forces.^33^ The outcomes of these responses are increased contractility, and a rise in intravascular volume which maintains the effective circulating volume, increases venous return and diastolic stress. In the functional state demonstrated by the volume-overloaded LMS an abnormal amount of preload is required for any meaningful force production (*Figure 3B*). This is not only chronically unsustainable but constrained *in vivo* on the lower end by hypoperfusion and on the upper end by pulmonary and systemic congestion.

Under this conceptual framework, as the harmful process iterates itself due to recurring cycles of decompensation,^33^ it is likely that not only the absolute force but also the sensitive working range of cardiac operation diminishes until the tissue is unable to respond to any stretch at all. In support of this, human papillary muscles from terminal heart failure, the last stage of disease, are unable to employ the Frank–Starling response.^34^ Furthermore, this idea is bolstered by increased compliance of volume-overloaded LMS (*Figure 3D*). Decreased passive tension has been shown in volume-overloaded rats^35^ and human DCM,^36^ and pathophysiologically means less resistance to ventricular filling. Thus, it may be a contributing intrinsic myocardial mechanism (in contrast to systemic mechanisms that cause a rise in intravascular volume), which allows the ventricle to reach the higher preload that is still sensitive to stretch.

The response to mechanical preload or afterload has been reported to be mediated by different signalling cascades.^2^ We found 380 and 291 genes that are expressed uniquely in pressure- or volume-overload but not the other. Volume-overload, which has been studied less in comparison to pressure-overload, involves activation of the Akt-mTOR-PI3K pathway,^37^^,^^38^ and this pathway’s activation shows a positive relationship with diastolic wall stress.^37^ At least 13 genes involved in the PI3K-Akt pathway were significantly altered in volume-overloaded LMS, including *Fgfr2, Igf1, Col6a6, Ngfr, Itga9, Ddit4, Itga3, Vegfa, Il3ra, Cdk6, Pdgfra, Hsp90b1*, and *Jak2* (*Table 1*).

**Table 1:** Selected significantly different genes in volume- and pressure-overload compared to physiological load

Gene		ΔVO		ΔPO
Cellular adhesion molecules				
Itga3		↑		n.s
Itga9		↓		n.s
Itgbl1		↑		n.s
rhoc		↑		↑
ankrd37		n.s		↓
ankrd52		n.s		↑
PI3K-AKT				
IGF		↑		n.s
FGFR2		↑		n.s
NGFR		↑		n.s
VEGFA		↑		n.s
PDGFRA		↑		n.s
JAK2		↑		n.s
NF-κB				
C3		n.s		↑
CXCL1		n.s		↓
BCL2L1		n.s		↓
CDKN21A		n.s		↓
STAT5A		n.s		↑

Many preclinical models of pressure-overload have identified inflammation as a dominant driver of the remodelling response.^39–41^ A critical regulator is NF-Kβ, a transcriptional factor that orchestrates expression of an array of immunomodulators and cytokines.^42^ Pathway analysis of pressure-overloaded LMS showed dysregulation of an extensive network of inflammatory genes, many of which lie downstream of NF-κβ (*Table 1*). Furthermore, chronic pressure-overload is known to induce changes in ECM including deposition of collagen.^43^ Collagen, fibrotic (e.g. TGFbr3), and other ECM regulatory (e.g. *Adamts2, Eln*) genes were significantly up-regulated in pressure-overloaded LMS suggesting ECM remodelling. However, type 1 collagen protein content was not (*Figure 5D*). This may be due to a number of reasons. Our *in vitro* model lacks neurohormonal regulation, which may be necessary for development of fibrosis.^44^ To that end, isolated fibroblasts cultured in silicone plates increase type III collagen synthesis within 24 h of cyclic stretch,^45^ however, LMS are a considerably different preparation where fibroblasts coexist with other cardiac cell populations and the natural ECM substrate of the heart. Indeed, when LMS are cultured under physiological preload but on fixed posts (i.e. infinite afterload) no collagen deposition is seen until Day 7 of culture.^46^ Longer culture timescales may be necessary for overt fibrosis.

In both overloaded groups, the endoplasmic reticulum protein processing pathway was functionally enriched. This included classical chaperons (*Hsp90, Hspa5*), and protein disulphide isomerases (*Pdia3, Pdia4*, and *Pdia6*). Impaired protein degradation can result in suboptimal protein quality, diminished contractile output, and a cycle of progressive maladaptive remodelling.^47^^,^^48^ Furthermore, multiple mechanotransduction genes, which initiate remodelling^38^ by converting early mechanical stimuli to biochemical signals were also significantly altered in the overloaded groups (*Table 1*).

All differentially expressed genes shared between the overloaded groups had the same direction of change compared to physiological load, except for α-actinin (*Acnt1*). *Actn1* is involved in eccentric and concentric myofibrillar remodelling^49^ and was decreased in pressure- and increased in volume-overload, respectively. However, protein content of α-actinin was not different between the overloaded groups; it is possible that this is a consequence of different proteolytic rates. Calpain is the Ca^2+^-dependent protease that degrades α-actinin.^50^ The gene expression of this was significantly up-regulated in volume-overloaded but not pressure-overloaded LMS. It is noteworthy that *Actn1* is up-regulated exclusively in human myocardium with depressed cardiac function,^50^ a state similar to that exhibited by volume-overloaded cultured LMS here.

The *Nppb* gene codes for brain natriuretic peptide (BNP), a hormone secreted by ventricular myocytes in response to stress. An unexpected finding of our study was therefore the relative decrease in *Nppb* expression in overloaded groups compared to physiological load. However, similar findings have been reported in cultured human LMS,^6^ and although a relatively good marker for HF, BNP levels are highly variable and even decrease during early disease.^51^ Furthermore, the natriuretic system has been suggested to not only mark but modulate remodelling and its dysregulation to potentially cause progressive dysfunction in the setting of chronic overload.^52^ Thus, a decreased BNP expression in overloaded LMS, of unknown mechanism and timepoint within culture, could put them at a disadvantage. In support of this, *Nppb* knockouts show an amplified hypertrophic phenotype in response to increased blood pressure,^53^ while reintroduction of the BNP receptor gene (*Npr1*) in mouse knockout models of hypertrophy decreases myocyte size.^54^

### 4.3 Limitations and future work

*In vivo*, the distinction between pressure- and volume-overload is less clear with the pathophysiology of many cardiac diseases involving features of both in a complex, dynamic, and non-linear manner.^17^ The work reported here describes experimental groups that reflect pure pressure- or volume-overload. However, the 3EWK parameters can be adjusted to simulate overlapping conditions. Likewise, in response to mechanical overload *in vivo*, there is up-regulation of multiple systemic compensatory mechanisms that operate in a feedback fashion. As these are missing here, the reported results must be interpreted within the context afforded by an *in vitro* model.

During culture, rat LMS were paced at 1 Hz, which is in contrast to the higher *in vivo* heart rates of a rat (5–6 Hz). This was done, as in most chronic *in vitro* culture studies, to reduce metabolic and O_2_ demands on the tissue.^6,8,7^ Finally, only one LMS can be cultured at a time in our current system. Development of cheaper technologies is needed to increase throughput, which is necessary for scarcely available tissue such as LMS from human donors and explanted hearts.

## 5. Conclusion

We have developed a novel platform and methodology to culture cardiac tissue *in vitro*, under finely tuned, physiological or pathological mechanical preload and afterload. We show proof of concept that by chronically imposing pathological load on LMS, cardiac pathophysiology can be recreated and studied in adult cardiac tissue *in vitro*. Our approach, which is complementary to *in vivo* experiments, will enable many applications including the study of remodelling for basic insight into mechanisms of disease, and the testing of new therapeutics.

## Supplementary material

Supplementary material is available at *Cardiovascular Research* online.

## Authors’ contributions

F.G.P. conceptualized and performed the experiments, wrote the code, analysed the data, and wrote the manuscript. R.N.T., W.K.A., S.M., and R.J.J. performed experiments, and edited the manuscript. K.X. analysed the data and edited the manuscript. S.E.H., F.P., and T.T. edited the manuscript. P.P.T. conceptualized the experiments, wrote the code, and edited the manuscript. C.M.T. conceptualized the experiments, analysed the data, and edited the manuscript.

### Acknowledgements

We would like to thank the Hannover Medical School Research Core Unit Genomics (RCUG) for the next-generation RNA sequencing. We would also like to thank Stephen Rothery from the Facility for Imaging by Light Microscopy (FILM) at Imperial College London for his help with confocal imaging.

**Conflict of interest**: T.T is the founder and shareholder of Cardior Pharmaceuticals GmbH. All other authors declare no conflicts of interest, financial or otherwise.

## Supplementary Material

cvab084_Supplementary_Data

## Notes

Translational perspective Mechanical overload is one of the principal drivers of pathological cardiac remodelling. In clinics, overload is divided into pressure-overload (e.g. aortic stenosis) and volume-overload (e.g. valvular regurgitation). Currently, most of our understanding of pathological remodelling in response to load comes from data obtained *in vivo*. However, *in vitro* cardiac models afford higher degrees of causality and are indispensable in increasing our understanding of the underlying mechanisms of load-induced remodelling. Yet, mechanical load is poorly recreated *in vitro*, and only distantly recapitulates the load imposed on the heart *in vivo*. This limits pathophysiological relevance and translation of findings from laboratory to clinics. Here we first developed a bioreactor to simulate the *in vivo* mechanical load *in vitro*. Then we cultured adult living myocardial tissue in the bioreactor and showed that we can recreate load-induced remodelling *in vitro*.
