# Supplementary material for: Remodelling of adult cardiac tissue subjected to physiological and pathological mechanical load in vitro
Source: Cardiovasc Res. 2021 Mar 16;118(3):814–27. doi: 10.1093/cvr/cvab084 (PMC8859636; doi:10.1093/cvr/cvab084)

Supplementary Material

Remodelling of adult cardiac tissue subjected to physiological and pathological mechanical load *in vitro*

Short title: Load-induced cardiac remodelling *in vitro*

Authors

Fotios G Pitoulis[1], Raquel Nunez-Toldra[1], Ke Xiao[2], Worrapong Kit-Anan[1], Saskia Mitzka[2], Richard J Jabbour[1], Sian E Harding[1], Filippo Perbellini[2], Thomas Thum[2,1], Pieter P de Tombe[3], Cesare M Terracciano[1,*]

Affiliations

1: National Heart and Lung Institute, Imperial College London, UK

2: Institute for Molecular and Translational Therapeutic Strategies, Hannover Medical School, DE, Germany

3: University of Illinois at Chicago, USA

Supplementary 1: Twitch force developed by LMS throughout culture

Twitch force was monitored continuously (i.e. every beat) by our bioreactor and logged every 5 min. Force developed by LMS is shown here with a 4 h sampling frequency to avoid clattering of data. Data are shown as mean + SEM; N = 6.

Supplementary 2: Sarcomere length-% stretch relationships from resting muscle length

The SL - % stretch relationship was determined using laser diffraction experiments and a linear regression was fitted to the data. This allowed us to stretch LMS to the desired preload based on resting muscle length. N =18 LMS/6 biological replicates.

Supplementary 3: Screenshot of custom time-varying elastance program for assessment of load-independent contractile state

The code iteratively fits a linear regression across each ms of the cardiac cycle for all fed loops. The slope of each linear regression, equal to elastance, $E_{(t)}$ is then plotted (far right panel), and maximum elastance, E_{max}, is automatically determined and saved in a .txt file.

Supplementary 4: Tissue media pH at end culture

The pH of the media bathing the LMS was measured at day 3 of culture. Data are shown as mean + SEM; N = 4-5.

Supplementary 5: Analysis method for cardiomyocyte dimensions

A representative figure from a pressure-overloaded LMS is shown twice. The first corresponds to the raw image. The second corresponds to the analysed image whereby a rectangle is drawn around 8 cardiomyocytes and used for determination of cell are length, and width. All analysis was done blinded by two independent reviewers.

Supplementary 6: MHC protein expression of cultured LMS

Western blots were used to quantify MHC protein content. There were no significant differences in MHC content between LMS. Data are shown as mean ± SEM; N = 5.

Supplementary 7: Western blot gels

Supplementary 1

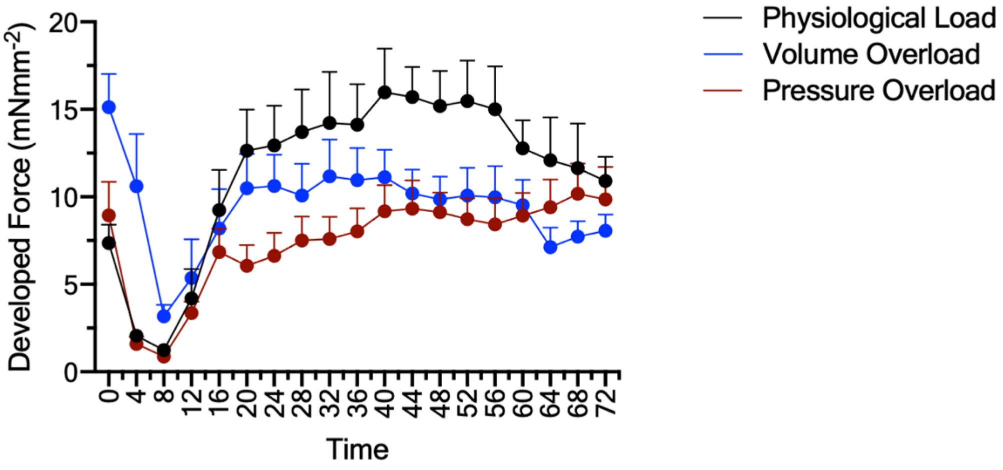

Supplementary 2

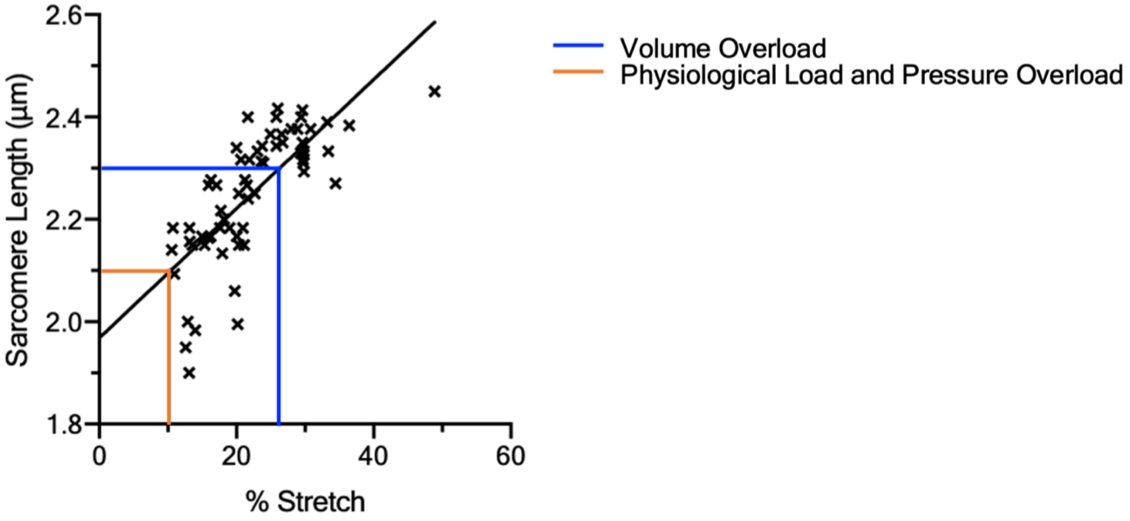

Supplementary 3

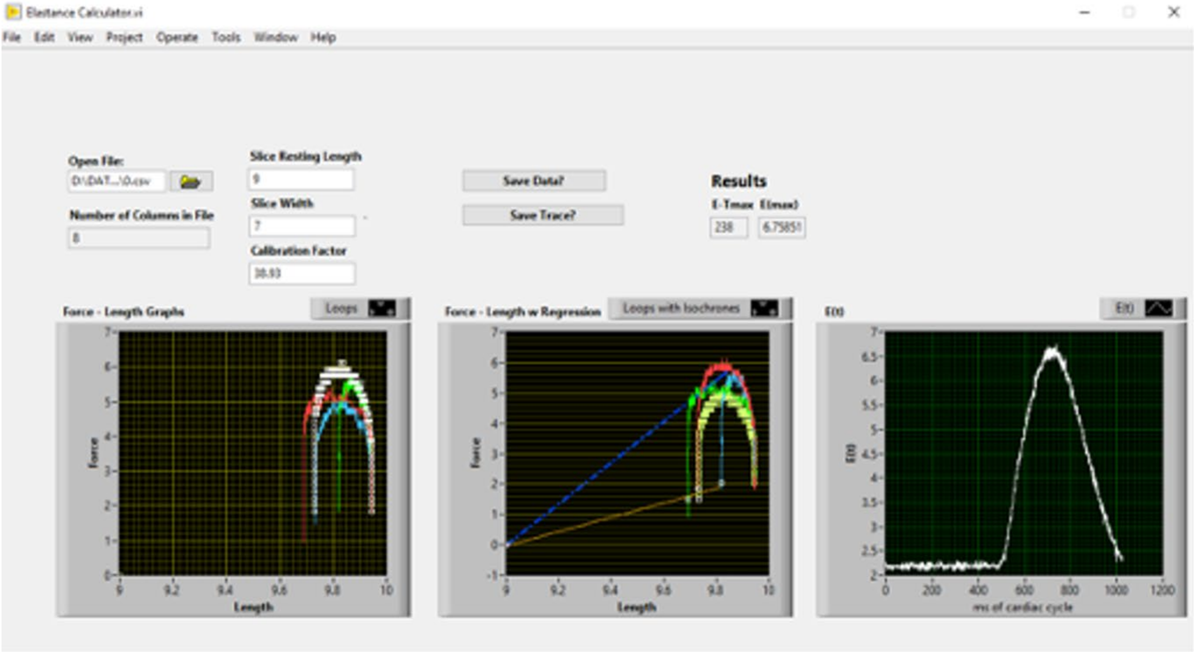

Supplementary 4

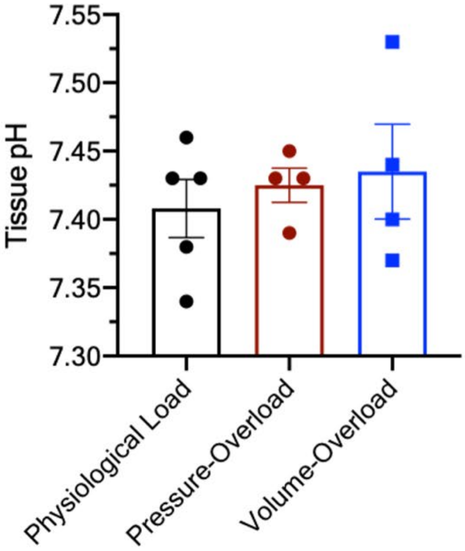

Supplementary 5A

1 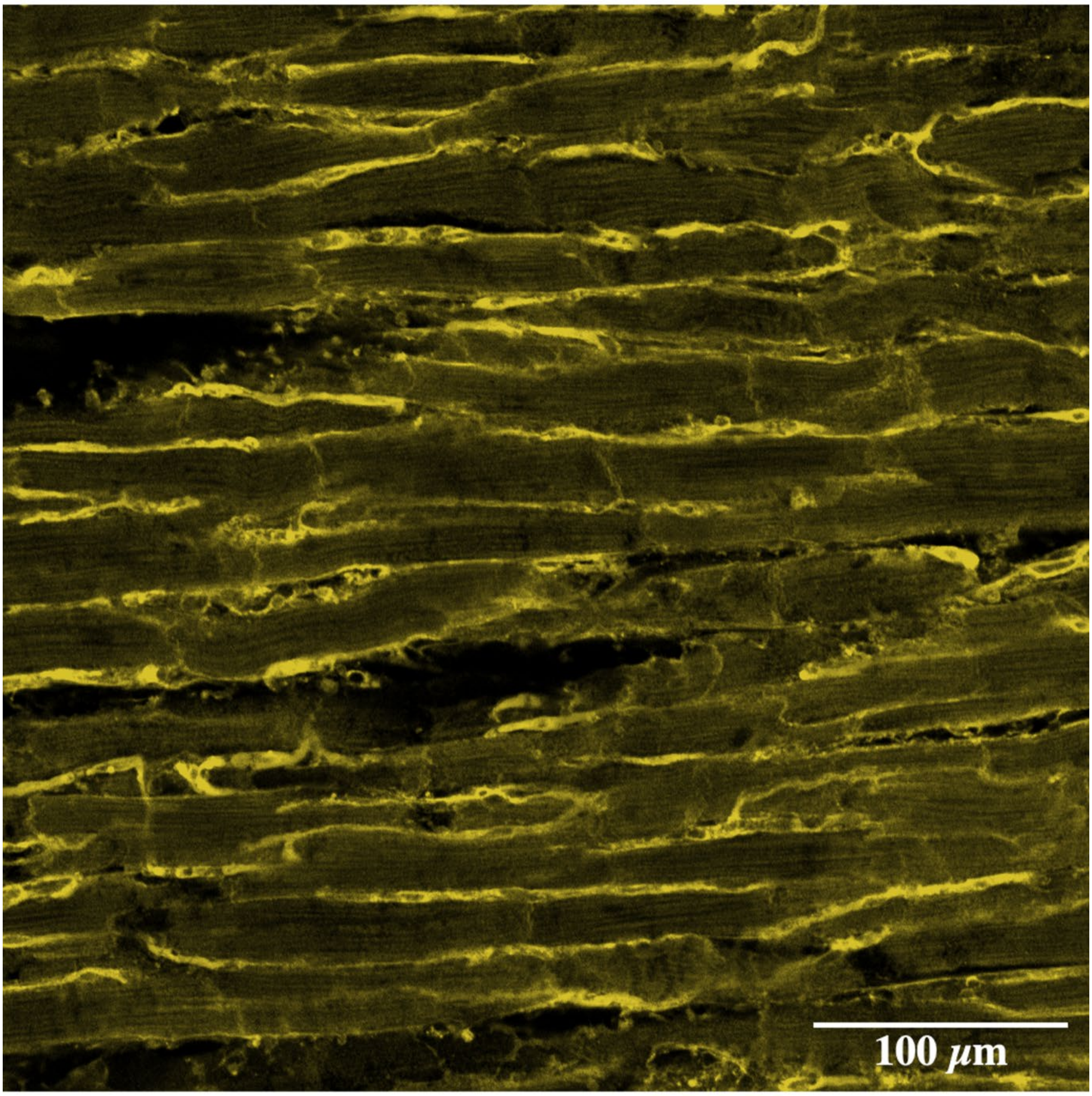

Supplementary 5B

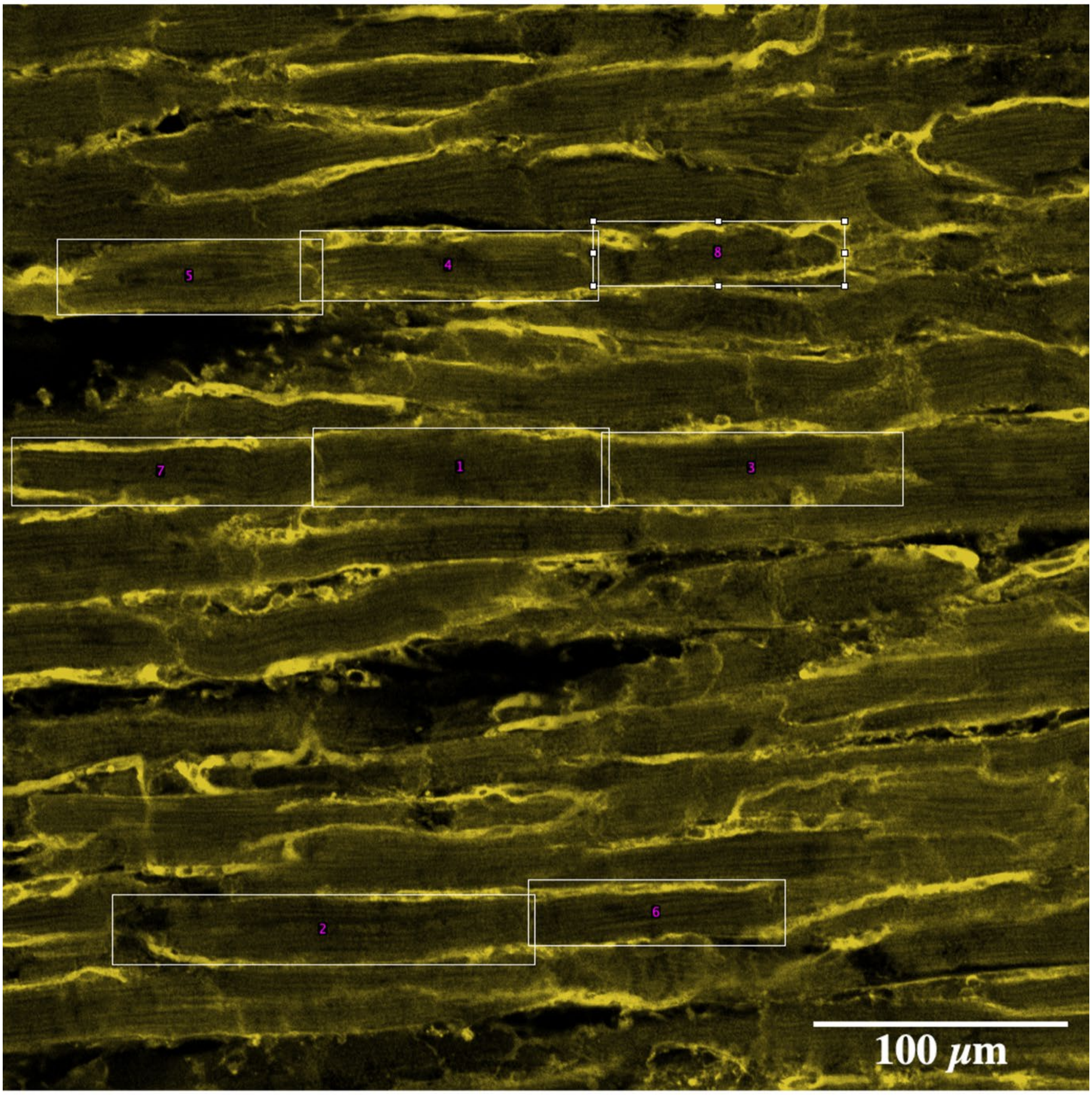

Supplementary 6

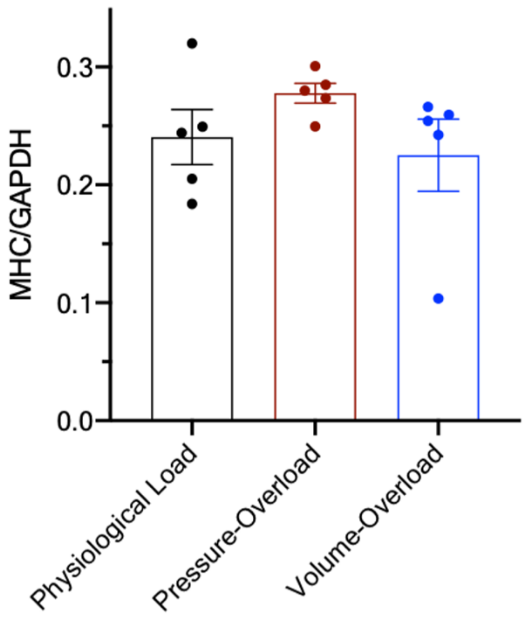

Supplementary 7

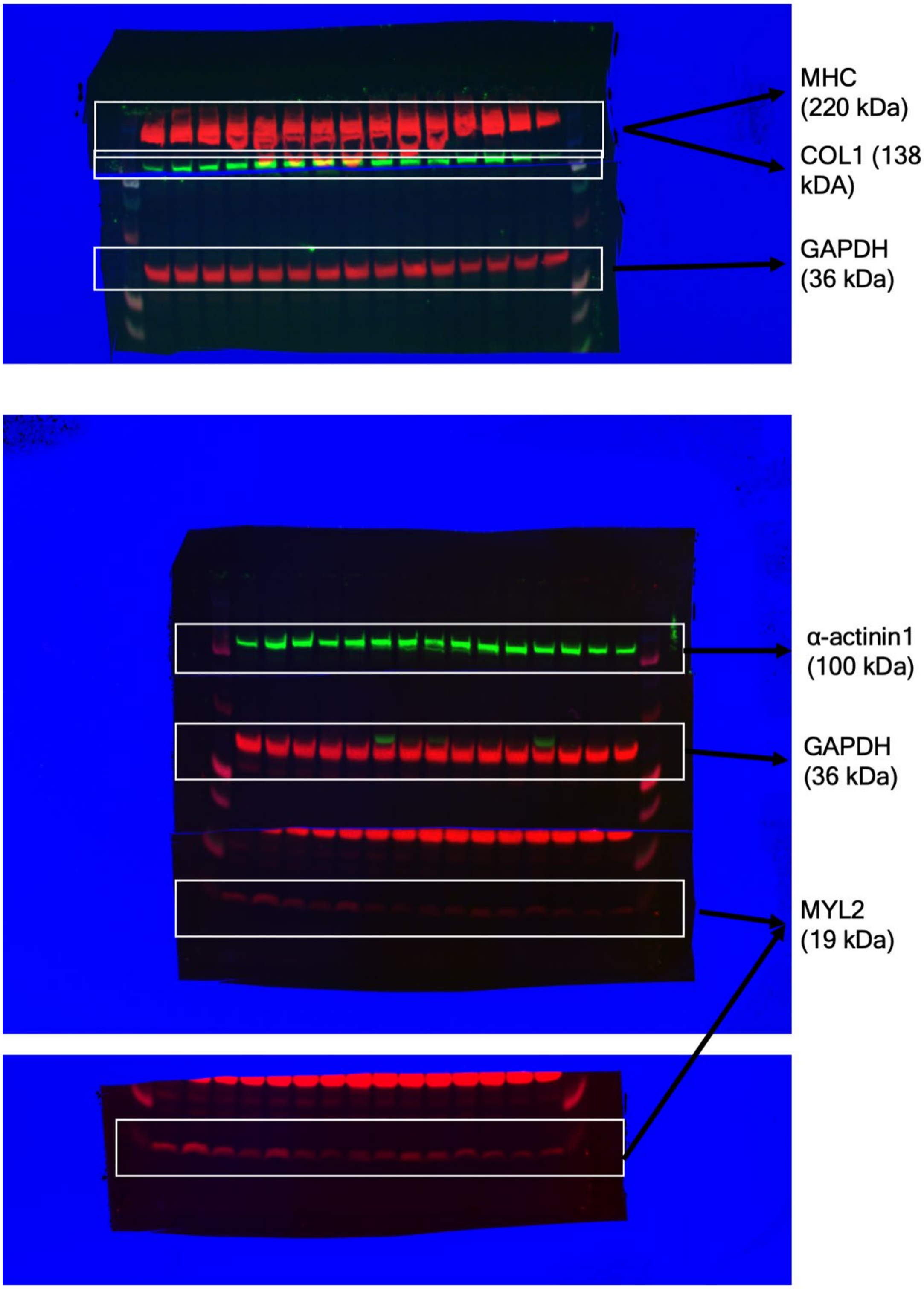

Supplement: cvab084_Supplementary_Data [file cvab084_supplementary_data.pdf]
